# Long-term exposure to particulate matter and risk of Alzheimer’s disease and vascular dementia in Korea: a national population-based Cohort Study

**DOI:** 10.1186/s12940-023-00986-9

**Published:** 2023-04-14

**Authors:** Jung-Im Shim, Garam Byun, Jong-Tae T. Lee

**Affiliations:** 1 College of Health Science, Korea University, Seoul, 02841 Republic of Korea; 2 Division of Healthcare Technology Assessment Research, National Evidence-based Healthcare Collaborating Agency, Seoul, 04933 Republic of Korea; 3 Interdisciplinary Program in Precision Public Health, Korea University, Seoul, 02841 Korea

**Keywords:** Particulate matter (PM_10_), Vascular dementia, Alzheimer’s disease, Dementia, Cohort

## Abstract

**Background:**

The prevalence of age-related neurodegenerative diseases has risen in conjunction with an increase in life expectancy. Although there is emerging evidence that air pollution might accelerate or worsen dementia progression, studies on Asian regions remains limited. This study aimed to investigate the relationship between long-term exposure to PM_10_ and the risk of developing Alzheimer’s disease and vascular dementia in the elderly population in South Korea.

**Methods:**

The baseline population was 1.4 million people aged 65 years and above who participated in at least one national health checkup program from the National Health Insurance Service between 2008 and 2009. A nationwide retrospective cohort study was designed, and patients were followed from the date of cohort entry (January 1, 2008) to the date of dementia occurrence, death, moving residence, or the end of the study period (December 31, 2019), whichever came first. Long-term average PM_10_ exposure variable was constructed from national monitoring data considering time-dependent exposure. Extended Cox proportional hazard models with time-varying exposure were used to estimate hazard ratios (HR) for Alzheimer’s disease and vascular dementia.

**Results:**

A total of 1,436,361 participants were selected, of whom 167,988 were newly diagnosed with dementia (134,811 with Alzheimer’s disease and 12,215 with vascular dementia). The results show that for every 10 µg/m^3^ increase in PM_10_, the HR was 0.99 (95% CI 0.98-1.00) for Alzheimer’s disease and 1.05 (95% CI 1.02–1.08) for vascular dementia. Stratified analysis according to sex and age group showed that the risk of vascular dementia was higher in men and in those under 75 years of age.

**Conclusion:**

The results found that long-term PM_10_ exposure was significantly associated with the risk of developing vascular dementia but not with Alzheimer’s disease. These findings suggest that the mechanism behind the PM_10_-dementia relationship could be linked to vascular damage.

**Supplementary Information:**

The online version contains supplementary material available at 10.1186/s12940-023-00986-9.

## Introduction

An increase in life expectancy leads to an increase in the prevalence of age-related neurodegenerative diseases [56]. Most of these diseases progress to dementia and are usually diagnosed when social and/or occupational functions cannot be performed because of acquired cognitive impairment [24]. According to the American Psychiatric Association Diagnostic and Statistical Manual (DSM-5), dementia, a major neurocognitive disorder, results from severe dysfunction in one or more cognitive domains including memory, language, visuospatial ability, and social/behavioral function [15, 24]. Dementia is often divided into two broad categories: neurodegenerative diseases such as Alzheimer’s disease and Parkinson’s disease, and non-neurodegenerative diseases such as vascular dementia [20]. According to the pathophysiological process, the clinical classification of dementia is divided into Alzheimer’s disease (50–70%), vascular dementia (20%), Lewy body dementia (5%), and frontotemporal dementia (5%) [15].

In 2016, approximately 47 million people worldwide were reported to suffer from dementia; this number is expected to triple to approximately 115.4 million by 2050 [47, 56]. In particular, the prevalence of dementia in the population aged 65 years and older is estimated to double every five years [26]. The cost of health services, including caring for people with dementia, is increasing, and patients’ families are burdened by physical, emotional, and financial stress [3, 15, 24].

Currently, there is no established cure for dementia, emphasizing the importance of prevention and early intervention [56]. While aging serves as the principal risk factor for dementia, it remains non-modifiable. Thus, identifying modifiable risk factors for dementia is crucial in order to effectively prevent and manage the risk of dementia within the population. Potential modifiable risk factors include apolipoprotein E (*APOE*) ε4, hypertension, obesity, smoking, diabetes, depression, cardiovascular disease, head injury, and social isolation. Some protective factors include physical activity, healthy diet, and non-steroidal anti-inflammatory drugs (NSAIDs) [15, 18, 24, 38, 43]. Environmental exposure is another risk factor that can be modified, and recent epidemiological studies have suggested that air pollution may accelerate or worsen dementia [5, 51]. In a recent systematic review, long-term exposure to PM_2.5_ was associated with the risk of dementia [19, 46, 58] and cognitive decline [57]. Other findings provided inconsistent evidence on the effects of PM_10_ and air pollution such as nitrogen oxides (NO_x_), nitrogen dioxide (NO_2_), or ozone (O_3_) exposure on dementia development and cognitive function [1, 59, 60].

Studies on air pollution and dementia remain limited in Asian cities [48]. In Korea, some studies have reported the effects of air pollution on cognitive function and Parkinson’s disease [31, 44, 52], and the Clinical Research Center for Dementia of South Korea (CREDOS) study cohort reported that PM_2.5_ exposure was exacerbated neuropsychiatric symptoms in people with cognitive impairment [32]. There was no PM_10_-dementia study in Korea, and this study is the first study to investigate the relationship between PM_10_ and dementia. Korea is a serious aging society, and age-related neurodegenerative diseases will put an economic burden [29]. This study aimed to determine the relationship between long-term exposure to PM_10_ and the risk of developing Alzheimer’s disease and vascular dementia in an older population using the National Health Insurance Service (NHIS).

## Methods

### Data source

The national health insurance service (NHIS) is a public database that covers the entire population of South Korea, and the population included in the data is over 50 million [50]. The NHIS, as the single insurer, covers 100% of the Korean population and consists of national health insurance (NHI) for both employees (70.4%) and self-employed insured individuals (26.8%), as well as medical aid (MA) beneficiaries (2.8%) in 2019 [27]. The NHIS database contains demographic and medical claims data, including types of medical care facilities, dates of visits, diagnosis codes, medical costs, procedures, prescribed drug information, and examinations. In addition, this database is linked to the death records of National Statistics Korea. The diagnostic records were coded using the International Statistical Classification of Diseases (ICD-10). This study was approved by the Institutional Review Board of Korea University, which waived the need for informed consent because only de-identified data were used (IRB code KUIRB-2021-0003-01).

### Study design and population

This was a nationwide, retrospective cohort study. The baseline population for our study consisted of individuals aged 65 years or older who participated in the national health screening program between 2008 and 2009. Patients were followed from the date of cohort entry to the date of dementia occurrence, death, moving residence, or the end of the study period (December 31, 2019), whichever came first. We assumed that individuals with only one health record during the study period were a result of administrative record errors, as this data source is secondary data for the national health insurance. Therefore, individuals with fewer than two health records documented between 2008 and 2019 were excluded. Additionally, the following patients were excluded: (1) those who were diagnosed with dementia or had a history of dementia-related drugs between 2005 and 2007; (2) those who died before the cohort entry date; (3) those for whom there was no information on covariates; and (4) those for whom there was no air pollution information at baseline.

We created two separate cohorts for Alzheimer’s disease and vascular dementia. The Alzheimer’s disease cohort consisted of Alzheimer’s disease and dementia-free groups, and the vascular dementia cohort consisted of vascular dementia and dementia-free groups.

### Outcome definition

Referring to a previous study [4], we identified an incident case of dementia up to the fifth diagnosis code. A dementia event was defined as ≥ 1 inpatient or 2 outpatient records in the neurology or psychiatry department and prescription of dementia-related medications (donepezil, rivastigmine, galantamine hydrobromide, and memantine). Dementia type was classified as Alzheimer’s disease (ICD-10: F00, G30), vascular dementia (ICD-10: F01), and others (ICD-10: F02-F03, F05.1, G31.1, G31.0, G31.8).

To detect incident cases of dementia, patients who developed dementia within one year of cohort entry were censored at the time of dementia onset in each subject. Moreover, several definitions were compared to validate the definition of outcome as follows: (1) restricted to primary and secondary diagnosis codes, presence of ≥ 1 inpatient or 2 outpatient diagnoses, and a prescription of dementia medication (strict definition); a case of dementia up to the fifth diagnosis code with (2) presence of ≥ 1 record of an inpatient or outpatient diagnosis, and a prescription of dementia medication; (3) presence of ≥ 1 inpatient or 2 outpatient diagnoses, or a prescription of dementia medication; and (4) presence of either a dementia diagnosis or a prescription of dementia medication.

### Exposure assessment

Air Korea (www.airkorea.or.kr) was used to collect data from the region-specific sites. The data were sent to the National Ambient Air Monitoring Information System (NAMIS), and these were confirmed and finalized by the National Institute of Environmental Research. If more than 75% of the data were complete, they were considered valid.

We obtained hourly concentrations of particles < 10 μm in diameter (PM_10_), nitrogen dioxide (NO_2_), sulfur dioxide (SO_2_), ozone (O_3_), and carbon monoxide (CO) at each monitoring site from 2008 to 2019. The study area included 137 districts (study population per district: 1,733 − 30,939, median size of districts: 73,512,354 km^2^) and 186 monitoring stations across South Korea (Supplementary Table 1), each of which had at least one monitoring site. If there were more than one monitoring station in a district, the average of the measurements was taken every hour within each district.

We then calculated district-specific daily 24-hour mean concentrations for PM_10_, NO_2_, and SO_2_and the daytime 8-hour [09:00–17:00] concentrations for O_3_and CO. We selected weekly averages of O_3_and CO concentrations to better represent outdoor exposure. If more than 6 h (25%) of the 24-hour measurements are missing, the concentration for that date was treated as missing. Next, we averaged the daily values over 12 months (all-season) for each district and calendar year. We also treated the values for a year as missing if more than 25% of the data were missing.

To calculate the time-varying exposure, district-specific yearly mean concentrations were assigned to individuals based on their residence in each calendar year. The time-varying exposures were defined as the 1-year averages. When the study population moved away from their residence, the moving date was assumed to be July 1, the year in which they moved.

### Covariates

Potential confounders were identified in the literature, including demographic variables (age, sex, body mass index (BMI)), socioeconomic factors (insurance premium), behavioral factors (smoking, drinking, physical activity), comorbidities (depression, traumatic brain injury, hypertension, diabetes mellitus, hyperlipidemia, coronary artery disease, cerebrovascular disease, atrial fibrillation, peripheral vascular disease, myocardial infarction, stroke, chronic obstructive pulmonary disease (COPD), chronic liver disease, chronic pulmonary disease, Charlson comorbidity index (CCI)), and ecological factors (Supplementary Table 2).

Ecological factors were collected from KOSIS (the Korean Statistical Information Service) at a province or district scale. The proportion of the elderly was calculated as the ratio of the population aged 65 years and older to the total population, and the proportion of basic livelihood security was the ratio of the number of recipients of basic livelihood security. The proportion of people without a high school diploma was defined as the population without a high school diploma, divided by those aged 25–64. Education data were available for 2005; therefore, the 2008–2009 study population’s education categories were based on the 2005 data. The ratio of the elderly and education was calculated as a single value for each district, and the proportion of basic livelihood security was calculated on the provincial scale.

### Statistical analysis

Extended Cox proportional hazard models were used with time-varying exposure to estimate hazard ratios (HR) for the long-term effects of PM_10_. As the exposure varied over the years, the dataset was prepared for analysis using the Andersen-Gill counting process. The counting process method had a yearly record for each participant, and records were generated until one of the following occurred first: dementia onset, death, moving, or ending of a cohort event. For example, one person with no events up to 2019 had 12 annual records. Furthermore, the association between the incidence of Alzheimer’s disease and vascular disease was estimated according to annual mean PM_10_ exposure.

The final models were adjusted for potential confounders, including sex, age, BMI, smoking, physical activity, insurance premium, and comorbidities, including depression, traumatic brain injury, diabetes mellitus, stroke, CCI, proportion of basic livelihood security recipients, and proportion of people with no high school diploma. All covariates were treated as fixed variables except for age. Age was assigned to time-dependent covariates. The results are reported as estimated HRs with 95% CIs per 10 µg/m^3^ increase in PM_10_. The effects of PM_10_ on dementia were estimated as both continuous and categorical variables, and average exposure levels were categorized into quartiles (Q1, < 42.9 µg/m^3^; Q2, 42.9–47.6 µg/m^3^; Q3, 47.7–53.3 µg/m^3^; Q4, ≥ 53.4 µg/m^3^). To test the exposure-response relationship between PM_10_ and dementia, we used a restricted natural cubic spline function with 4 knots.

Subgroup analyses were conducted to estimate the potential effect modifications according to sex (male and female), age (< 75 or ≥ 75 years), stroke (yes or no), depression (yes or no), brain injury (yes or no), and diabetes mellitus (yes or no). Sensitivity analyses were performed to explore the robustness of the results to the exposure time window, potential outcome misclassifications, and confounding factors. First, the annual average concentrations of PM_10_ were computed for the previous three and five years (3-year, and 5-year moving). Second, alternative definitions of outcomes were applied. Third, we excluded participants who died during the study period, as death could be considered as a competing risk for dementia. Fourth, regional indicators (16 province levels) as alternative ecological factor were applied. Finally, we adjusted the models for other pollutants including NO_2_, SO_2_, O_3_, and CO. All analyses were performed using SAS Enterprise Guide 7.1 for Windows and R version 4.1.0. The statistical significance level was set at p = 0.05.

## Results

**Fig. 1:**
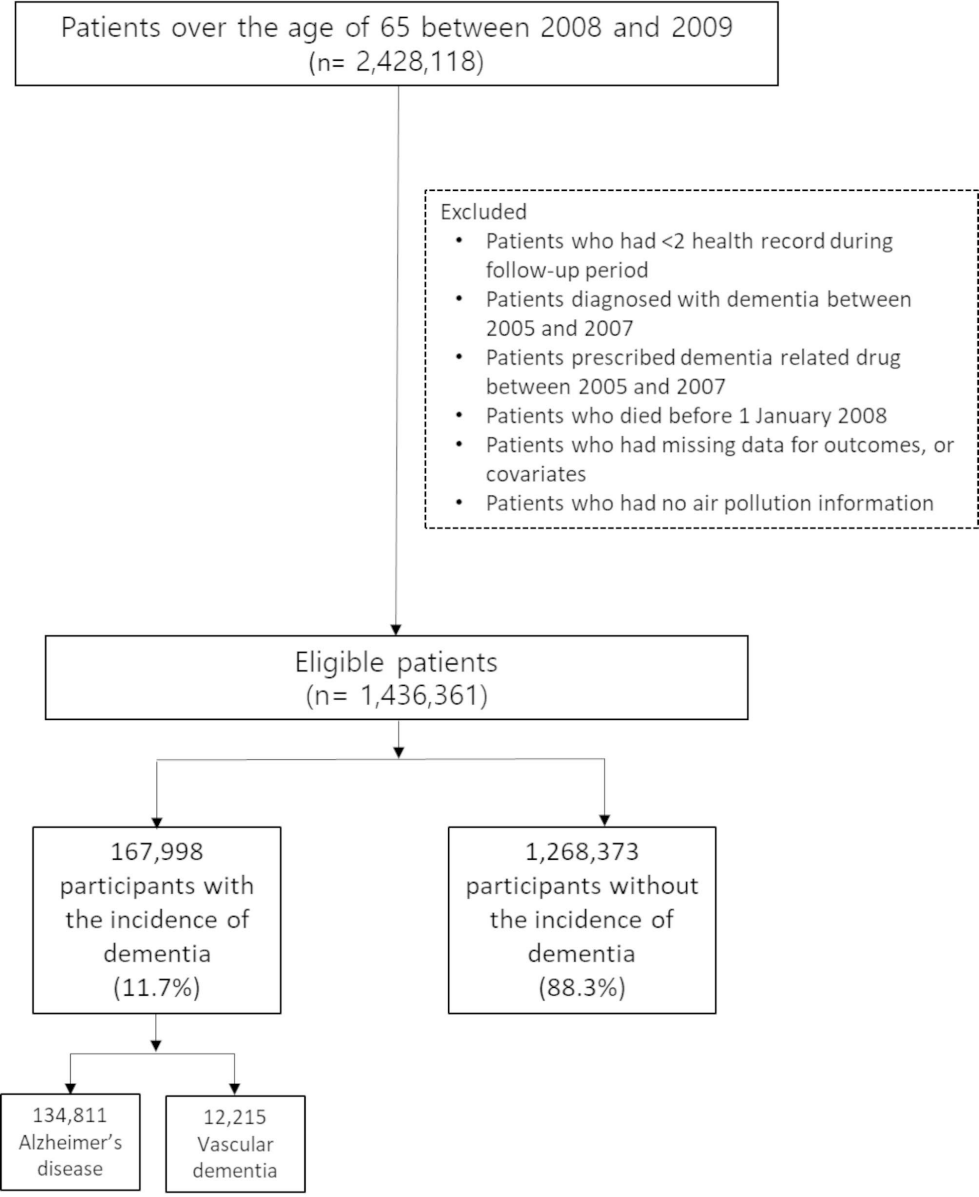
Flow chart of the inclusion process for the dementia cohort

A total of 1,436,361 participants were followed after excluding 991,757 individuals by predefined exclusion criteria. A total of 167,988 participants were newly diagnosed with dementia during the study period, accounting for 11.7% of the study population. Among them, 134,811 and 12,215 individuals were identified as having Alzheimer’s disease and vascular dementia, respectively, accounting for 80.3% and 7.3% of the total number of dementia cases, respectively (Fig. 1).

Of the 1,436,361 participants, 53.4% were female, 20.8% were aged 75 years or older, and 62.8% were overweight or obese. The majority of participants were non-smokers (75.5%), non-drinkers (78.0%), and exercised less (41.7%). Compared to all subjects, patients with Alzheimer’s disease and vascular dementia had a higher prevalence of comorbidities, particularly depression, hypertension, cerebrovascular disease, stroke, and CCI. The mean follow-up time was 8.6 years and the mean concentration of PM_10_ was 48.4 µg/m^3^ (Table 1).

**Table 1:** Baseline characteristics of Alzheimer’s disease, vascular dementia, and non-dementia cohort for 2008–2019

		All			Alzheimer’s disease			Vascular dementia	
		N = 1,436,361			N = 134,811			N = 12,215	
**Sex**									
	Male	669,452	(46.6)		51,361	(38.1)		5,544	(45.4)
	Female	766,909	(53.4)		83,450	(61.9)		6,671	(54.6)
**Age**									
	Mean ± SD	70.9	± 4.9		72.8	± 5.0		72.4	± 4.8
	64–69	660,135	(46.0)		38,348	(28.5)		3,812	(31.2)
	70–74	478,516	(33.3)		51,394	(38.1)		4,692	(38.4)
	75–79	209,352	(14.6)		31,689	(23.5)		2,697	(22.1)
	≥ 80	88,358	(6.2)		13,380	(9.9)		1,014	(8.3)
**Insurance premium**									
	Q1	260,168	(18.1)		21,430	(15.9)		1,996	(16.3)
	Q2	204,095	(14.2)		18,959	(14.1)		1,698	(13.9)
	Q3	327,911	(22.8)		30,411	(22.6)		2,768	(22.7)
	Q4	644,187	(44.8)		64,011	(47.5)		5,753	(47.1)
**Region** ^*^									
	7 Metropolitan	701,345	(48.8)		56,489	(41.9)		4,826	(39.5)
	9 Province	735,016	(51.2)		78,322	(58.1)		7,389	(60.5)
**BMI**									
	< 23	534,276	(37.2)		53,849	(39.9)		4,682	(38.3)
	23-<25	377,439	(26.3)		34,251	(25.4)		3,123	(25.6)
	≥ 25	524,646	(36.5)		46,711	(34.7)		4,410	(36.1)
**Smoking**									
	Non	1,084,788	(75.5)		108,045	(80.2)		9,270	(75.9)
	Past	175,085	(12.2)		13,069	(9.7)		1,397	(11.4)
	Present	176,488	(12.3)		13,697	(10.2)		1,548	(12.7)
**Drinking**									
	< 1/week	1,120,964	(78.0)		111,217	(82.5)		9,722	(79.6)
	1–2/week	167,038	(11.6)		11,931	(8.9)		1,201	(9.8)
	≥ 3/week	148,359	(10.3)		11,663	(8.7)		1,292	(10.6)
**Physical activity**									
	None	598,686	(41.7)		63,421	(47.0)		5,855	(47.9)
	1–2/week	191,296	(13.3)		16,578	(12.3)		1,529	(12.5)
	3–4/week	175,165	(12.2)		15,059	(11.2)		1,256	(10.3)
	≥ 5/week	471,214	(32.8)		39,753	(29.5)		3,575	(29.3)
**Comorbidities**									
Depression		78,957	(5.5)		11,266	(8.4)		971	(8.0)
Traumatic brain injury		12,147	(0.8)		1,441	(1.1)		167	(1.4)
Hypertension		746,606	(52.0)		74,506	(55.3)		7,517	(61.5)
Diabetes mellitus		345,783	(24.1)		37,356	(27.7)		3,796	(31.1)
Hyperlipidemia		350,502	(24.4)		35,055	(26.0)		3,463	(28.4)
Coronary artery disease		178,569	(12.4)		18,684	(13.9)		1,779	(14.6)
Cerebrovascular disease		154,026	(10.7)		19,030	(14.1)		2,494	(20.4)
Atrial fibrillation		23,154	(1.6)		2,397	(1.8)		295	(2.4)
Peripheral vascular disease		123,727	(8.6)		14,042	(10.4)		1,288	(10.5)
Myocardial infarction		15,754	(1.1)		1,589	(1.2)		164	(1.3)
Stroke		110,242	(7.7)		13,836	(10.3)		1,984	(16.2)
COPD		186,585	(13.0)		19,378	(14.4)		1,758	(14.4)
Chronic liver disease		113,267	(7.9)		11,341	(8.4)		1,055	(8.6)
Chronic pulmonary disease		392,344	(27.3)		39,989	(29.7)		3,552	(29.1)
CCI	0	556,618	(38.8)		44,703	(33.2)		3,706	(30.3)
	1–2	575,048	(40.0)		55,114	(40.9)		4,883	(40.0)
	≥ 3	304,695	(21.2)		34,994	(26.0)		3,626	(29.7)
Proportion of elderly (mean ± SD)		9.8	± 3.2		10.2	± 3.5		9.9	± 3.5
Proportion of the basic livelihood security recipient (mean ± SD)		3.2	± 1.3		3.4	± 1.4		3.3	± 1.3
Proportion of people with no high school diploma (mean ± SD)		21.4	± 7.6		22.6	± 7.9		22.3	± 7.8
Follow-up (year) (mean ± SD)		8.6	± 4.1		7.4	± 3.0		6.6	± 2.8
PM_10_(µg/m^3^) (mean ± SD)		48.4	± 7.7		46.0	± 7.0		47.2	± 7.1

BMI, body mass index; SD, standard deviation; COPD, chronic obstructive pulmonary disease; CCI, Charlson comorbidity index.

^*^The region was categorized into seven metropolitans and nine rural areas. The metropolitan areas include Seoul, Busan, Incheon, Gwangju, Daegu, Daejeon, and Ulsan. The rural areas included Gyeonggi, Gangwon, Chungbuk, Chungnam, Jeonbuk, Jeonnam, Gyeongbuk, Gyeongnam, and Jeju.

In the univariate model, the HR for Alzheimer’s disease with an increase in 10 µg/m^3^ of PM_10_ was 0.94 (95% CI 0.94–0.95) and that for vascular dementia was 1.03 (95% CI 1.01–1.06). After adjustment for demographic variables, behavioral factors, socioeconomic factors, comorbidities and ecologic variables, the HR was 0.99 (95% CI 0.98–1.00) for Alzheimer’s disease and 1.05 (95% CI 1.02–1.08) for vascular dementia for every 10 µg/m^3^ increase in PM_10_. In the case of vascular dementia, the risk of dementia increased in Q3 compared with the lowest quartile (Q1) of PM_10_, but it was not statistically significant in Q4 (Table 2). The exposure-response curves between PM_10_ and dementia showed a linear relationship up to a specific concentration but became constant at specific concentrations (Supplementary Fig. 1).

**Table 2:** Hazard ratio of Alzheimer’s disease and vascular dementia per 10 µg/m^3^ increase in PM_10_

	Alzheimer’s disease			Vascular dementia	
	(No. of events = 134,811)			(No. of events 0= 12,215)	
	HR^*^	(95% CI)		HR^*^	(95% CI)
Crude	0.94	(0.94, 0.95)		1.03	(1.01, 1.06)
Adjusted^*^	0.99	(0.98, 1.00)		1.05	(1.02, 1.08)
Quintile					
Q1(< 42.9)	1.00	(reference)		1.00	(reference)
Q2(42.9 ≤ 47.7)	1.00	(0.98, 1.01)		1.10	(1.05–1.16)
Q3(47.7 ≤ 53.4)	1.01	(1.00, 1.03)		1.11	(1.06, 1.17)
Q4(≥ 53.4)	0.99	(0.97, 1.00)		1.04	(0.98, 1.10)

^*^Adjusted for sex, age as a time-varying covariate, BMI, smoking, physical activity, insurance premium, comorbidities including depression, traumatic brain injury, stroke, diabetes mellitus, CCI, proportion of basic livelihood security recipients, and proportion of people without a high school diploma

BMI, body mass index; CCI, Charlson comorbidity index; HR, hazard ratio; CI, confidence interval.

In stratification analyses by sex and age, positive and statistically significant association between PM_10_ and vascular dementia were observed in male and those younger than 75 years of age (HR [95% CI] per 10 µg/m^3^ for male:1.08 [1.03–1.12], < 75 years:1.07 [1.03–1.11]). In the subgroup with or without stroke, the HRs of vascular dementia incidence associated with PM_10_ exposure were 0.96 (95% CI 0.89-1.02) and 1.07 (95% CI 1.04–1.10), respectively (Table 3).

**Table 3:** Hazard ratio of dementia per 10 µg/m^3^ increase in PM_10,_ according to sex, age, and comorbidities

	Alzheimer’s disease			Vascular dementia	
	HR^*^	(95% CI)		HR^*^	(95% CI)
**Sex**					
Male	1.00	(0.98, 1.01)		1.08	(1.03, 1.12)
Female	0.98	(0.97, 1.00)		1.02	(0.99, 1.06)
**Age group**					
< 75	1.01	(1.00, 1.02)		1.07	(1.03, 1.11)
≥ 75	0.96	(0.95, 0.98)		1.01	(0.96, 1.06)
**Stroke**					
No	0.99	(0.98, 1.00)		1.07	(1.04, 1.10)
Yes	1.01	(0.98, 1.03)		0.96	(0.89, 1.02)
**Depression**					
No	0.99	(0.98, 1.00)		1.05	(1.02, 1.08)
Yes	0.99	(0.96, 1.02)		1.03	(0.94, 1.13)
**Brain injury**					
No	0.99	(0.98, 1.00)		1.05	(1.02, 1.08)
Yes	0.94	(0.86, 1.02)		1.06	(0.84, 1.35)
**Diabetes mellitus**					
No	0.99	(0.98, 1.00)		1.05	(1.02, 1.09)
Yes	0.99	(0.95, 1.01)		1.04	(0.99, 1.09)

^*^Adjusted for sex, age as a time-varying covariate, BMI, smoking, physical activity, insurance premium, comorbidities including depression, traumatic brain injury, stroke, diabetes mellitus, CCI, proportion of basic livelihood security recipients, and proportion of people without a high school diploma.

BMI, body mass index; CCI, Charlson comorbidity index; HR, hazard ratio; CI, confidence interval.

In the sensitivity analyses, the robustness of the results was confirmed by exposure time windows, outcome definitions, exclusion of subjects who died, and adjustment for the indicator variable of region. According to the co-pollutant analyses, our estimated HRs for PM_10_showed consistent, significant associations on vascular dementia after adjusting for 1-year moving average concentrations of other pollutants (Table 4).

**Table 4:** Sensitivity analysis for hazard ratio of dementia per 10 µg/m^3^ increase in PM_10_

	Alzheimer’s disease			Vascular dementia		
	No. of event	HR^*^	(95% CI)	No. of event	HR^*^	(95% CI)
**Main analysis (1-year moving average)**	134,811	0.99	(0.98, 1.00)	12,215	1.05	(1.02, 1.08)
**Exposure time window**						
3-year moving average	134,811	0.98	(0.97, 0.99)	12,215	1.04	(1.01, 1.07)
5-year moving average	134,811	0.97	(0.96, 0.99)	12,215	1.02	(0.99, 1.05)
**Outcome definition**						
Restricted to secondary diagnosis and treatment for dementia	117,014	0.99	(0.98, 1.00)	9,128	1.06	(1.03, 1.10)
≥ 1 inpatient or outpatient diagnosis and treatment for dementia	149,579	1.00	(0.99, 1.00)	13,090	1.05	(1.03, 1.08)
≥ 1 inpatient or 2 outpatient diagnoses or treatment for dementia	126,323	0.98	(0.97, 0.99)	16,363	1.06	(1.03, 1.08)
Dementia diagnosis or treatment for dementia	137,080	0.99	(0.98, 1.00)	18,790	1.06	(1.03, 1.08)
**Excluded participants with death**	134,732	0.99	(0.98, 1.00)	12,205	1.05	(1.02, 1.08)
**Region variable**						
Region indicator (16 province)^**^	134,811	0.96	(0.95, 0.97)	12,215	1.08	(1.05, 1.12)
EDU + RECIP + Region indicator^†^	134,811	0.96	(0.95, 0.97)	12,215	1.08	(1.05, 1.12)
**Multi-pollutant models** ^‡^						
PM_10_ + SO_2_^‡^	134,811	1.00	(0.99, 1.00)	12,215	1.07	(1.04, 1.10)
PM_10_ + SO_2_ + NO_2_^‡^	134,811	1.00	(0.99, 1.01)	12,215	1.07	(1.04, 1.10)
PM_10_ + SO_2_ + NO_2_ + O_3_^‡^	134,811	1.00	(0.99, 1.01)	12,215	1.07	(1.04, 1.10)
PM_10_ + SO_2_ + NO_2_ + O_3_ + CO^‡^	134,811	1.00	(0.99, 1.01)	12,215	1.07	(1.04, 1.10)

^*^Adjusted for sex, age as a time-varying covariate, BMI, smoking, physical activity, insurance premium, comorbidities including depression, traumatic brain injury, stroke, diabetes mellitus, CCI, proportion of basic livelihood security recipients, and proportion of people without a high school diploma.

^**^Adjusted for sex, age as a time-varying covariate, BMI, smoking, physical activity, insurance premium, comorbidities including depression, traumatic brain injury, stroke, diabetes mellitus, CCI, region.

^**^Region indicator was classified into 16 provinces including Seoul, Busan, Daegu, Incheon, Gwangju, Ulsan, Daejeon, Gyeonggi, Gangwon, Chungbuk, Chungnam, Jeonbuk, Jeonnam, Gyeongbuk, Gyeongnam, and Jeju.

^†^Adjusted for sex, age as a time-varying covariate, BMI, smoking, physical activity, insurance premium, comorbidities including depression, traumatic brain injury, stroke, diabetes mellitus, CCI, proportion of basic livelihood security recipients, proportion of people with no high school diploma, and region indicator.

^‡^Adjusted for sex, age as a time-varying covariate, BMI, smoking, physical activity, insurance premium, comorbidities including depression, traumatic brain injury, stroke, diabetes mellitus, CCI, proportion of basic livelihood security recipients, proportion of people with no high school diploma, and gaseous pollutants specified in each model. The HRs represent estimates of PM_10_ adjusting for other pollutants.

BMI, body mass index; CCI, Charlson comorbidity index; EDU, proportion of people with no high school diploma; RECIP, proportion of basic livelihood security recipients. HR, hazard ratio; CI, confidence interval.

## Discussion

This study investigated the association between long-term exposure to PM_10_ and risk of dementia in Korea from 2008 to 2019. The results revealed that long-term exposure to PM_10_increased the risk of developing vascular dementia but was not positively associated with Alzheimer’s disease. Stratified analysis according to sex and age group showed that the risk of vascular dementia was higher in male and in those under 75 years of age. The associations were robust to various sensitivity analyses, including exposure time window and outcome definition. These findings provide evidence of a potentially significant implication of exposure to PM_10_ on vascular dementia.

Previous studies have suggested that long-term exposure to PM_2.5_significantly increases the risk of dementia [9, 19, 30, 51, 53]. Also, exposure to PM_2.5_was observed to increase the risk of Alzheimer’s disease and vascular dementia [7, 34, 48]. In contrast to consistent evidence supporting the adverse effects of PM_2.5_on cognitive decline, the research on PM_10_has been limited and the results have not been conclusive [59]. The inconsistency in the results for PM_10_may be attributed to several factors, including study populations, exposure measurement methods, and statistical analysis methods. Despite the potential for heterogeneity in estimates, the effect estimate (PM_10_estimate per 10 µg/m^3^) from the prior Rome study [8] (for vascular dementia, HR = 1.06, 95% CI 1.02–1.10; for Alzheimer’s disease, HR = 0.95, 95% CI 0.91–0.99) was line with our findings (for vascular dementia, HR = 1.05, 95% CI 1.02–1.08; for Alzheimer’s disease, HR = 0.99, 95% CI 0.98–1.00).

The most common mechanisms are believed to be inflammation and oxidative stress, and PM can affect the central nervous system (CNS) through direct and indirect pathways [6, 41]. Fine particles less than 2.5 μm (PM_2.5_) or smaller particles can enter directly the nasal olfactory pathway, where the inhaled particles can enter systemic circulation or penetrate through cellular membranes to reach the brain [6]. Another pathway is the peripheral immune system, where PM_10_and PM_2.5_induce pro-inflammatory signals in the immune system triggering a cytokine response that transmits inflammation to the brain [6, 48]. In vivo study, long-term exposure to PM_10_was associated with an increased risk of amyloid-ß (Aβ) positivity with regard to CNS pathologies [33]. Another study reported that exposure to PM_10_and PM_2.5_was associated with higher concentrations of cerebrospinal fluid, a biomarker of Alzheimer’s disease [2].

PM-induced systemic inflammation, especially interleukin-1 alpha (IL-1α) and tumor necrosis factor-alpha (TNF-α), is likely to cause stroke [40], lung disease, [54], cardiovascular disease [49] and neurodegenerative diseases [13, 14, 45]. Vascular dementia is known to causes damage to brain function owing to vascular lesions, and these vascular disorders frequently occur in the brain of the elderly [55]. In addition, cardiovascular disease (CVD) associated with chronic PM exposure leads to be a pathway that can induce cerebrovascular dysregulation through vasoconstriction [35].

Stratified analysis according to sex and age group showed that the risk of vascular dementia was higher in male and in those under 75 years of age. Studies investigating gender differences in cognitive health have reported that women may be more susceptible to Alzheimer’s disease due to several factors, including longer life expectancy and higher disease morbidity [28, 42]. According to the ILSA study, men have been found to have a significantly higher risk of developing vascular dementia compared to women [16]. Another study reported that before the age of 79, vascular dementia was more prevalent among men, but after the age of 85, it became more prevalent among women [39]. The relationship between age, gender, and vascular dementia risk may be complex and influenced by risk factors, such as body size, smoking, diabetes, obesity, myocardial infarction, and stroke. For instance, some comorbidity, such as diabetes, and obesity may have a more significant negative effect on women than men, while some cardiovascular risk factors, such as hyperlipidemia and myocardial infarction, may be a greater influence on men [21]. Taken together, our findings suggest that the relationship between PM_10_exposure and vascular dementia risk may be influenced by gender and age differences, as well as other risk factors. Further research is needed to fully understand the complex interplay of these factors and their impact on cognitive health.

We conducted stratified analysis on depression, brain damage, diabetes, and stroke to determine if there was a difference in effect size based on the presence of comorbidities. In particular, we examined the difference in effect size between patients with and without stroke, which showed a significant difference. Previous studies have used effect modifiers and mediators via CVD to establish an association between air pollution and dementia [22, 25]. Although no clear effect modification was identified, our study found a potentially higher incident risk of vascular dementia related to PM_10_exposure among participants without stroke (HR = 1.07, 95% CI 1.03–1.11) compared to those with stroke (HR = 0.96, 95% CI 0.89–1.02). One potential explanation for this observation could be selection bias, as it is possible that patients with stroke may die before developing dementia. Nevertheless, further research is necessary to fully understand the underlying mechanisms.

To test the potential non-linear relationship between PM_10_and dementia, we assessed the shape of the exposure-response curve by using a restricted natural cubic spline function with 4 knots. In our curves, the slope for PM_10_-vascular dementia was steeper at concentrations lower than 50 µg/m^3^, and the slopes seed to flatter at high ranges. Some previous studies also have suggested a violation of the log-linearity assumption for particulate matter effects on population mortality and morbidity [17, 36, 37]. One possible explanation could be that populations living in areas with high exposure to PM may develop an adaptive response, resulting in smaller estimates of exposure changes per unit [37]. Also, when the PM concentration is high, there is a possibility that the actual individual’s exposure may change due to public health policy interventions such as wearing masks and reducing outdoor activities [11]. Nonetheless, it is noteworthy that the risk of vascular dementia may still be present even at PM_10_concentrations below 50 µg/m^3^, which is the national annual standard for PM_10_.

This study has some limitations. First, there is a possibility of misclassification the onset of dementia. Owing to the nature of the administrative dataset, some dementia patients might not had been diagnosed. Also, the dataset included both incident and progressive cases among people diagnosed with dementia according to ICD-10 codes. To reduce outcome misclassification, predefined criteria were defined, and the robustness of the results was tested. The dementia subtypes in this study were Alzheimer’s disease (80.3%) and vascular dementia (7.3%), which showed a distribution similar to a previous study conducted in Korea (Alzheimer’s disease:86.1% in 2016; vascular dementia:10.6% in 2016) [12].

Second, our exposure assessment was based on district-level address information at the baseline, which did not completely reflect personal exposure. Although assigning an average exposure to each individual at a fixed monitoring site would introduce a Berkson error, the error is not expected to significantly affect the measurements and estimates [23]. In addition, there was no information regarding the distance from the road. In a cohort study, living close to a major road was associated with an increased risk of dementia, particularly Alzheimer’s disease [10].

Previous studies have mostly reported the effect of PM_2.5_ exposure on dementia, and PM_2.5_ may be a more appropriate indicator in terms of biological mechanism. Although PM_2.5_ exposure estimates were not available because the data were established in 2015, the correlation between PM_2.5_ and PM_10_ concentrations was high (r > 0.73) within our data (2015–2019). When other air pollutants, including NO_2_, SO_2_, O_3_, and CO, were added to the model, PM_10_ estimates were robust.

One of the strengths of this study is the first study to examine the long-term effect of PM_10_ on dementia, in a representative, a large population-based cohort using data from a nationwide database collected over 15 years. This provided significant statistical power to detect the association between PM_10_ and dementia. Second, we were able to determine the residential mobility of the study population, which could have reduced the exposure measurement error. Third, we included various covariates in the model to minimize the residual confounding. Using medical records, pre-existing diseases were identified at baseline and behavioral variables were obtained in connection with health examination data. In addition, the models were adjusted for ecological variables such as the proportion of basic livelihood security recipients and people with no high school diploma.

## Conclusions

In this large population-based cohort, long-term exposure to PM_10_ was associated with a higher incidence of vascular dementia but not Alzheimer’s disease. In addition, the risk of vascular dementia was higher in men and those under 75 years of age. These results may contribute to understanding the relationship between air pollution and dementia by providing information on populations vulnerable to air pollution. This study may implicate the evidence that exposure to air pollution may be more associated with dementia, especially in terms of vascular damage.

## Electronic supplementary material

Below is the link to the electronic supplementary material.

Supplementary Material 1

## Data Availability

The data that support the findings of this study was used under license for the current study, and hence not publicly available. Data codebooks and syntaxes used for the statistical analyses are however available from the authors upon request.

## Abbreviations

BMI: Body mass index
CCI: Charlson comorbidity index
CI: Confidence interval
COPD: Chronic obstructive pulmonary disease
CVD: Cardiovascular disease
EDU: Proportion of people with no high school diploma
HR: Hazard ratio
ICD-10: International Statistical Classification of Diseases
NHIS: National Health Insurance Service
SD: Standard deviation
PM: Particulate matter
RECIP: Proportion of basic livelihood security recipients
