# Supplementary material for: Long-term exposure to particulate matter and risk of Alzheimer’s disease and vascular dementia in Korea: a national population-based Cohort Study
Source: Environ Health. 2023 Apr 14;22:35. doi: 10.1186/s12940-023-00986-9 (PMC10105439; doi:10.1186/s12940-023-00986-9)
Supplement: Supplementary file 1 — Supplementary Material 1 [file 12940_2023_986_MOESM1_ESM.docx]

**Supplementary Table 1. Study areas of 16 provinces and 137 district-level regions for PM_10_ exposure data**

| Province | Districts | Study population (n) | Area (㎡) |
| --- | --- | --- | --- |
| Seoul | Jongno-gu | 6,271 | 23,912,936 |
|  | Jung-gu | 5,149 | 9,960,262 |
|  | Yongsan-gu | 8,887 | 21,866,145 |
|  | Seongdong-gu | 11,226 | 16,859,899 |
|  | Dongdaemun-gu | 14,887 | 14,215,806 |
|  | Jungnang-gu | 14,309 | 18,495,584 |
|  | Seongbuk-gu | 16,010 | 24,576,989 |
|  | Gangbuk-gu | 13,831 | 23,600,441 |
|  | Dobong-gu | 14,259 | 20,651,004 |
|  | Nowon-gu | 17,938 | 35,439,209 |
|  | Eunpyeng-gu | 16,655 | 29,710,522 |
|  | Seodaemun-gu | 12,832 | 17,626,389 |
|  | Mapo-gu | 13,541 | 23,850,081 |
|  | Yangcheon-gu | 12,650 | 17,405,693 |
|  | Gangseo-gu | 14,969 | 41,452,201 |
|  | Guro-gu | 12,839 | 20,119,104 |
|  | Gumcheon-gu | 7,841 | 13,020,482 |
|  | Yeongdeungpo-gu | 13,352 | 24,548,211 |
|  | Dongjak-gu | 14,392 | 16,355,018 |
|  | Gwanak-gu | 17,477 | 29,568,314 |
|  | Seocho-gu | 9,498 | 46,981,621 |
|  | Gangnam-gu | 11,651 | 39,497,507 |
|  | Songpa-gu | 15,648 | 33,877,057 |
|  | Gangdong-gu | 11,577 | 24,590,492 |
| Busan | Jung-gu | 2,846 | 2,829,149 |
|  | Yeongdo-gu | 8,699 | 14,199,917 |
|  | Busanjin-gu | 18,236 | 29,667,096 |
|  | Dongnae-gu | 10,868 | 16,631,810 |
|  | Nam-gu | 13,302 | 26,820,380 |
|  | Buk-gu | 9,572 | 39,373,195 |
|  | Haeundae-gu | 15,237 | 51,540,907 |
|  | Saha-gu | 12,027 | 41,770,659 |
|  | Geumjeong-gu | 10,310 | 65,277,383 |
|  | Gangseo-gu | 2,779 | 181,500,580 |
|  | Yeonje-gu | 9,200 | 12,102,001 |
|  | Suyeong-gu | 8,338 | 10,218,180 |
|  | Sasang-gu | 8,028 | 36,104,593 |
|  | Gijang-gun | 4,515 | 218,316,025 |
| Daegu | Jung-gu | 4,090 | 7,056,240 |
|  | Dong-gu | 15,003 | 182,150,280 |
|  | Seo-gu | 9,456 | 17,319,408 |
|  | Nam-gu | 8,415 | 17,431,453 |
|  | Buk-gu | 14,832 | 93,987,602 |
|  | Suseong-gu | 15,556 | 76,535,524 |
|  | Dalseong-gun | 7,305 | 426,865,105 |
| Incheon | Jung-gu | 3,126 | 140,365,958 |
|  | Dong-gu | 3,048 | 7,197,170 |
|  | Yeonsu-gu | 5,943 | 56,192,485 |
|  | Namdong-gu | 10,796 | 57,449,150 |
|  | Bupyeong-gu | 15,020 | 32,004,527 |
|  | Gyeyang-gu | 7,426 | 45,567,499 |
|  | Seo-gu | 8,544 | 118,489,132 |
|  | Ganghwa-gun | 6,535 | 411,420,329 |
| Gwangju | Dong-gu | 5,971 | 49,314,065 |
|  | Seo-gu | 9,645 | 47,755,979 |
|  | Nam-gu | 8,715 | 60,990,032 |
|  | Buk-gu | 15,801 | 120,270,544 |
|  | Gwangsan-gu | 8,417 | 222,782,562 |
| Daejeon | Dong-gu | 10,792 | 136,670,767 |
|  | Jung-gu | 12,057 | 62,178,952 |
|  | Seo-gu | 15,405 | 95,318,145 |
|  | Yuseong-gu | 6,512 | 176,605,773 |
|  | Daedeok-gu | 6,575 | 68,729,951 |
| Ulsan | Jung-gu | 5,235 | 37,006,962 |
|  | Nam-gu | 6,267 | 73,512,354 |
|  | Dong-gu | 2,611 | 36,074,153 |
|  | Buk-gu | 2,662 | 157,361,696 |
|  | Ulju-gun | 7,939 | 758,372,513 |
| Gyeonggi-do | Suwon Jangan-gu | 6,573 | 33,342,085 |
|  | Suwon Gwonseon-gu | 6,278 | 47,179,157 |
|  | Suwon Paldal-gu | 5,860 | 12,856,945 |
|  | Seongnam Sujeong-gu | 7,830 | 45,453,256 |
|  | Seongnam Jungwon-gu | 7,139 | 26,412,623 |
|  | Seongnam Bundang-gu | 12,006 | 69,759,501 |
|  | Uijeongbu-si | 13,151 | 81,547,881 |
|  | Anyang Manan-gu | 8,076 | 36,545,855 |
|  | Anyang Dongan-gu | 8,683 | 21,938,176 |
|  | Bucheon-si | 20,596 | 53,453,519 |
|  | Gwangmyeong-si | 9,034 | 38,529,060 |
|  | Pyeongtaek-si | 13,388 | 458,255,093 |
|  | Dongducheon-si | 3,589 | 95,667,273 |
|  | Ansan Sangnok-gu | 7,094 | 57,991,984 |
|  | Ansan Danwon-gu | 5,790 | 98,417,756 |
|  | Goyang Deogyang-gu | 11,508 | 165,591,578 |
|  | Goyang Ilsan-gu | 15,698 | 102,520,602 |
|  | Gwacheon-si | 1,733 | 35,870,116 |
|  | Guri-si | 5,408 | 33,332,882 |
|  | Namyangju-si | 17,387 | 458,144,214 |
|  | Osan-si | 2,965 | 42,706,071 |
|  | Siheung-si | 7,435 | 139,683,554 |
|  | Gunpo-si | 7,398 | 36,416,592 |
|  | Uiwang-si | 3,649 | 54,034,758 |
|  | Hanam-si | 4,197 | 92,987,872 |
|  | Yongin-si | 25,328 | 591,235,776 |
|  | Paju-si | 13,702 | 673,880,294 |
|  | Icheon-si | 7,743 | 461,470,642 |
|  | Anseong-si | 7,917 | 553,465,426 |
|  | Gimpo-si | 7,973 | 276,601,527 |
|  | Hwaseong-si | 12,481 | 699,411,815 |
|  | Yangju-si | 6,524 | 310,469,902 |
|  | Pocheon-si | 7,897 | 826,986,435 |
| Gangwon-do | Chuncheon-si | 12,879 | 1,116,417,910 |
|  | Wonju-si | 14,369 | 868,251,130 |
|  | Gangneung-si | 13,192 | 1,040,753,600 |
|  | Donghae-si | 4,610 | 180,304,698 |
|  | Samcheok-si | 5,470 | 1,187,805,137 |
| Chungcheongbuk-do | Cheongju-si | 30,939 | 940,995,451 |
|  | Chungju-si | 11,502 | 983,618,696 |
|  | Jecheon-si | 8,248 | 882,766,071 |
| Chungcheongnam-do | Cheonan-si | 14,960 | 636,150,973 |
|  | Asan-si | 8,937 | 542,776,132 |
|  | Seosan-si | 9,333 | 742,179,548 |
|  | Dangjin-si | 10,875 | 705,528,041 |
| Jeollabuk-do | Jeonju-si | 23,846 | 206,016,741 |
|  | Gunsan-si | 12,415 | 397,454,458 |
|  | Iksan-si | 15,082 | 506,574,852 |
|  | Jeongeup-si | 9,967 | 693,097,194 |
|  | Namwon-si | 7,740 | 752,183,179 |
| Jeollanam-do | Mokpo-si | 8,885 | 51,656,909 |
|  | Yeosu-si | 13,542 | 512,259,113 |
|  | Suncheon-si | 12,019 | 910,948,821 |
|  | Gwangyang-si | 6,045 | 464,134,692 |
|  | Yeongam-gun | 5,608 | 612,484,981 |
| Gyeongsangbuk-do | Pohang Nam-gu | 8,520 | 393,994,642 |
|  | Pohang Buk-gu | 10,089 | 736,573,142 |
|  | Gyeongju-si | 17,468 | 1,324,890,516 |
|  | Gimcheon-si | 11,305 | 1,009,918,173 |
|  | Andong-si | 12,657 | 1,522,125,408 |
|  | Gumi-si | 9,646 | 615,305,568 |
|  | Yeongju-si | 9,380 | 670,101,796 |
| Gyeongsangnam-do | Changwon-si | 29,623 | 749,030,515 |
|  | Jinju-si | 14,134 | 712,903,526 |
|  | Gimhae-si | 11,866 | 463,522,923 |
|  | Yangsan-si | 7,613 | 485,617,219 |
|  | Hadong-gun | 6,427 | 674,910,207 |
| Jeju | Jeju-si | 15,275 | 978,721,709 |
|  | Seogwipo-si | 8,520 | 871,557,015 |

**Supplementary Table 2. Definition of comorbidities**

| Comorbidities (ICD-10) |  |
| --- | --- |
| Depression | F32, F33 |
| Traumatic brain injury | S020, S021, S027, S029, S060–S069 |
| Hypertension | I10-I13, I15 |
| Diabetes mellitus | E10–E14 |
| Hyperlipidemia | E780–E785 |
| Coronary artery disease | I20–I25 |
| Cerebrovascular disease | G45, G46, I60–I69, I71 |
| Atrial fibrillation | I48 |
| Peripheral vascular disease | I739, I790, R02, Z958, Z959 |
| Myocardial infarction | I21, I22 |
| Stroke | G45, H341, I60–I64 |
| COPD | J41-J44 |
| Chronic liver disease | B15-B19, C22, K70, K73, K74 |
| Chronic pulmonary disease | J40-J47, J60–J67, J684, J701, J703, J841, J920, J961, J982, J983 |

COPD, chronic obstructive pulmonary disease

Supplementary Table 3. Baseline characteristics of study population for 2008-2019

|  |  | All | | Dementia | | No dementia | |
| --- | --- | --- | --- | --- | --- | --- | --- |
|  |  | N=1,436,361 | | N=167,988 | | N=1,268,373 | |
| **Sex** | Male | 669,452 | (46.6) | 65,898 | (39.2) | 603,554 | (47.6) |
|  | Female | 766,909 | (53.4) | 102,090 | (60.8) | 664,819 | (52.4) |
| **Age** | mean±SD | 70.88 | ±4.92 | 72.77 | ±4.98 | 70.63 | ±4.86 |
|  | 64-69 | 660,135 | (46.0) | 48,008 | (28.6) | 612,127 | (48.3) |
|  | 70-74 | 478,516 | (33.3) | 63,935 | (38.1) | 414,581 | (32.7) |
|  | 75-79 | 209,352 | (14.6) | 39,335 | (23.4) | 170,017 | (13.4) |
|  | 80- | 88,358 | (6.2) | 16,710 | (10.0) | 71,648 | (5.7) |
| **Insurance premium** | Q1 | 260,168 | (18.1) | 26,753 | (15.9) | 233,415 | (18.4) |
|  | Q2 | 204,095 | (14.2) | 23,530 | (14.0) | 180,565 | (14.2) |
|  | Q3 | 327,911 | (22.8) | 37,847 | (22.5) | 290,064 | (22.9) |
|  | Q4 | 644,187 | (44.8) | 79,858 | (47.5) | 564,329 | (44.5) |
| **Region** | 7 Metropolitan | 701,345 | (48.8) | 70,758 | (42.1) | 630,587 | (49.7) |
|  | 9 Province | 735,016 | (51.2) | 97,230 | (57.9) | 637,786 | (50.3) |
| **BMI** | <23 | 534,276 | (37.2) | 66,947 | (39.9) | 467,329 | (36.8) |
|  | 23-<25 | 377,439 | (26.3) | 42,794 | (25.5) | 334,645 | (26.4) |
|  | ≥25 | 524,646 | (36.5) | 58,247 | (34.7) | 466,399 | (36.8) |
| **Smoking** | Non | 1,084,788 | (75.5) | 133,689 | (79.6) | 951,099 | (75.0) |
|  | past | 175,085 | (12.2) | 16,723 | (10.0) | 158,362 | (12.5) |
|  | present | 176,488 | (12.3) | 17,576 | (10.5) | 158,912 | (12.5) |
| **Drinking** | <1/week | 1,120,964 | (78.0) | 138,010 | (82.2) | 982,954 | (77.5) |
|  | 1-2/week | 167,038 | (11.6) | 15,115 | (9.0) | 151,923 | (12.0) |
|  | ≥3/week | 148,359 | (10.3) | 14,863 | (8.9) | 133,496 | (10.5) |
| **Physical activity** | None | 598,686 | (41.7) | 79,035 | (47.1) | 519,651 | (41.0) |
|  | 1-2/week | 191,296 | (13.3) | 20,880 | (12.4) | 170,416 | (13.4) |
|  | 3-4/week | 175,165 | (12.2) | 18,554 | (11.0) | 156,611 | (12.4) |
|  | ≥5/week | 471,214 | (32.8) | 49,519 | (29.5) | 421,695 | (33.3) |
| **Depression** |  | 78,957 | (5.5) | 13,966 | (8.3) | 64,991 | (5.1) |
| **Traumatic brain injury** | | 12,147 | (0.8) | 1,855 | (1.1) | 10,292 | (0.8) |
| **Hypertension** |  | 746,606 | (52.0) | 94,038 | (56.0) | 652,568 | (51.5) |
| **Diabetes mellitus** | | 345,783 | (24.1) | 47,335 | (28.2) | 298,448 | (23.5) |
| **Hyperlipidemia** |  | 350,502 | (24.4) | 44,145 | (26.3) | 306,357 | (24.2) |
| **Coronary artery disease** | | 178,569 | (12.4) | 23,502 | (14.0) | 155,067 | (12.2) |
| **Cerebrovascular disease** | | 154,026 | (10.7) | 24,765 | (14.7) | 129,261 | (10.2) |
| **Atrial fibrillation** | | 23,154 | (1.6) | 3,111 | (1.9) | 20,043 | (1.6) |
| **Peripheral vascular disease** | | 123,727 | (8.6) | 17,474 | (10.4) | 106,253 | (8.4) |
| **Myocardial infarction** | | 15,754 | (1.1) | 2,046 | (1.2) | 13,708 | (1.1) |
| **Stroke** |  | 110,242 | (7.7) | 18,219 | (10.9) | 92,023 | (7.3) |
| **COPD** |  | 186,585 | (13.0) | 24,248 | (14.4) | 162,337 | (12.8) |
| **Chronic liver disease** | | 113,267 | (7.9) | 14,183 | (8.4) | 99,084 | (7.8) |
| **Chronic pulmonary disease** | | 392,344 | (27.3) | 49,702 | (29.6) | 342,642 | (27.0) |
| **CCI** | 0 | 556,618 | (38.8) | 55,195 | (32.9) | 501,423 | (39.5) |
|  | 1-2 | 575,048 | (40.0) | 68,494 | (40.8) | 506,554 | (39.9) |
|  | ≥3 | 304,695 | (21.2) | 44,299 | (26.4) | 260,396 | (20.5) |
| **Proportion of elderly** (mean±SD) | | 9.78 | ±3.16 | 10.13 | ±3.44 | 9.73 | ±3.12 |
| **Proportion of the basic livelihood security recipient** (mean±SD) | | 3.16 | ±1.32 | 3.39 | ±1.39 | 3.13 | ±1.30 |
| **Proportion of people with no high school diploma** (mean±SD) | | 21.37 | ±7.57 | 22.42 | ±7.88 | 21.23 | ±7.52 |
| **Follow-up (year) (mean ± SD)** | | 8.6 | ±4.1 | 7.2 | ±3.0 | 8.7 | ±4.2 |
| **PM_10_(µg/m^3^) (mean ± SD)** | | 48.4 | ±7.7 | 46.2 | ±7.1 | 48.4 | ±7.7 |

BMI, body mass index; SD, standard deviation; COPD, chronic obstructive pulmonary disease; CCI, Charlson comorbidity index.

^*^The region was categorized into seven metropolitans and nine rural areas. The metropolitan areas include Seoul, Busan, Incheon, Gwangju, Daegu, Daejeon, and Ulsan. The rural areas included Gyeonggi, Gangwon, Chungbuk, Chungnam, Jeonbuk, Jeonnam, Gyeongbuk, Gyeongnam, and Jeju.

| 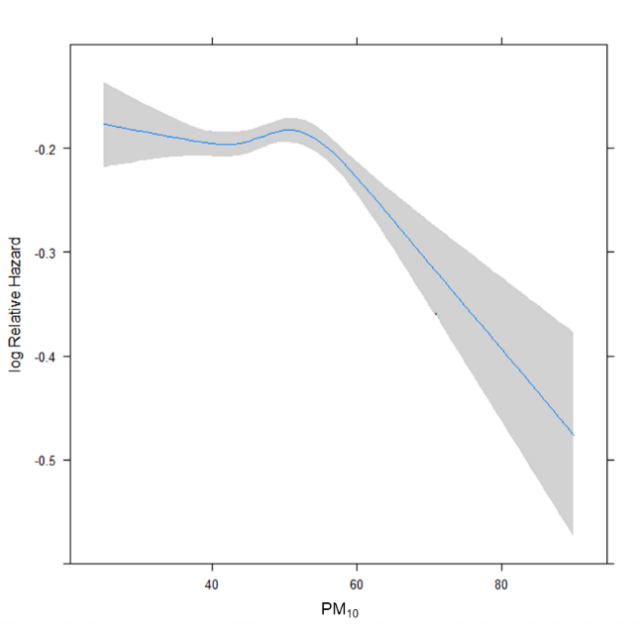 | 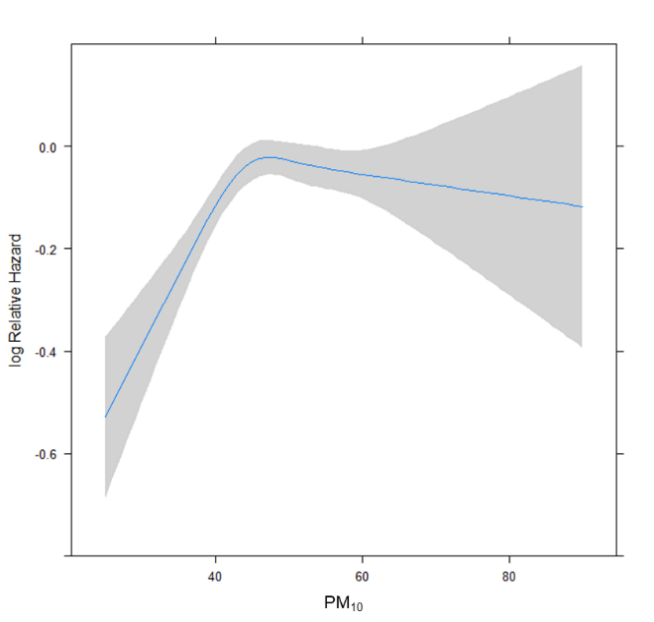 |
| --- | --- |
| **<Alzheimer’s Disease>** | **<Vascular Dementia>** |

Supplementary Figure 1. Exposure-response relationship between PM_10_ and dementia type
